# Echoes of the Month

**Published:** 1923-06

**Authors:** 


### ECHOES OF THE MONTH.

### OPEN-AIR PLAY CENTRES.

At a recent meeting of the Manchester and Salford Invalid Children's Aid Association, Dr. A. A. Mumford suggested the establishment of open-air playground centres in the parks for ailing children. Many nervous children threatened with minor chorea and emotional instability, aggravated by home conditions, were sent to the Association for convalescent treatment which it was unable to provide. If a few open-air playground centres could be established in the parks, where the children, instead of being placed under formal school discipline of rigid educational standards and subjected to the restraints of indoor school, could be happily employed in following organised occupations suitable to camp life under adequate supervision, not only would a large amount of suffering be averted, but the money spent by the educational authorities would be spent to much better advantage and the number of incompetent and ailing dependents on society materially diminished.

### THE EVOLUTION OF MAN.

Lecturing at the Royal Institution Sir Arthur Keith said that definite evidence existed that changes were taking place in the human body. From remains accumulated in the Museum of the Royal College of Surgeons, it was possible to show that there had been definite modifications in the formation of the human jaw, of the placing of the front teeth, of the character of the nose, with the result that the tendency was towards the production of a long and narrow face. There were marked changes also in the nature of thigh and leg bones, though the stature did not appear to have altered in the last four thousand years. As to whether the brain was or was not increasing, no definite answer could be given because sufficient skulls were not available for research, and the fact to-day was that not one person in fifty used their brain to half of its real capacity, most persons having more brains than they knew what to do with. The average stature of a man, 5 ft. 6 in., is the same as in the neolithic period.

### DISEASES TREATED BY POISON GAS.

The New York correspondent of "The Times" states that experiments conducted by the Army Medical Corps have shown the extraordinary efficacy of poison gases for the treatment of certain diseases ranging from simple colds to paresis. The experiments followed on the accidental discovery during the war that workers in the Edgewood Arsenal, near Baltimore, were immune from influenza in rooms in which chlorine gas was made. Guinea pigs inoculated with the germs of tuberculosis when exposed to mustard gas failed to contract the disease. Dr. A. S. Loevenhart, of the University of Wisconsin, who is co-operating with the Medical Corps, has a record of forty-two persons committed to asylums for paresis who were treated with Lewisite gas. Twenty-one have been discharged cured, and seven others give promise of recovery. As the result of experiments it is believed to be practicable to introduce small quantities of chlorine into schoolrooms, factories, and other crowded places as a protection against epidemics.

### A NEW TREATMENT FOR LEPROSY.

In Burma and Siam there grows a tree—the Taraktogenos Kurzii—which bears a tawny orange-like fruit. At the core of this fruit are a number of large seeds from which an oil known as chaulmoogra is obtained. For some years this oil has been used in the treatment of leprosy, but its usefulness has been lessened because it upsets digestion. Sir Leonard Rogers, Dr. Ernest Muir, the Leprosy Research Worker of the Calcutta School of Tropical Medicine, Dr. Wilson, of Kwangju, Korea, and others have been for some time experimenting with chaulmoogra and similar preparations. Certain other oils have also been found effective—for instance, those obtained from the Hydnocarpus Wightiana, a tree which grows freely in Burma, and the Hydnocarpus Anthelmintica, of South China. The active principles of the oil must be liberated and the ethyl esters thus obtained are injected intra-muscularly or intro-venously. A little caustic soda added to the oil yields sodium chaulmoograte; pour some hydrochloric acid on this sodium chaulmoograte and free chaulmoogric acid is obtained; if alcohol is now added to this chaulmoogric acid, ethyl chaulmoograte is liberated—the ethyl esters which are miscible with blood. After repeated injections the nodules are dispersed, the lesions disappear, and the bacilli are apparently destroyed once and for all. Those who are engaged in this work are careful not to speak of having "cured" lepers, but (the *Manchester Guardian* says) there are large numbers of cases that are now free from all the symptoms of leprosy.

### INFLUENCE OF SOIL ON THE TEETH.

Mr. Millican, L.D.S., in the annual report of the School Medical Officer for Norfolk (Dr. J. T. C. Nash) draws attention to the much larger percentage of "abnormal jaws in damp and low-lying places where the soil consists of clay, marl, or other impervious beds." The reason he gives is that such localities "lead to catarrhs and nasal obstruction, which it is generally agreed are likely to lead on to maldevelopment of the floor of the nose and roof of the mouth." He finds a much greater percentage of abnormal jaws in the eastern part of the county, which includes Broadland and the great spread of clay in Mid and South Norfolk, than in West Norfolk, where the chalk ridge exercises such a potent influence. The broad distinction between west and east is not, however, always justified, for the west includes part of Fenland and Marshland and a considerable number of villages on boulder clay, while local patches of glacial and Norwich Crag sand and gravel are used for the sites of many villages in the east.

### PROGRESS OF SANITATION IN AMERICA.

Dr. B. B. Bagly, of West Point, Virginia, has been giving to the Medical Society of Virginia some account of the sanitary conditions of West Point as recently as 1909. The City Fathers boasted of having the healthiest town in the State. "But let us see how very insanitary it was. Not a dwelling in town was completely screened. The negroes and poorer people had no screens at all. Every dwelling in town had an old-fashioned open privy. No thought had ever been given to draining the marshes to rid the town of malarial mosquitoes; and the chief dairyman of the town spent a part of every night cleaning out the open privies. The contract for cleaning these was let to the lowest bidder and the dairyman took the job at a very low figure, as he needed the refuse to fertilise his dairy farm land. He did his scavenger work late at night or early in the morning and went directly from that work to milking his cows, bottling and delivering milk. This was done with the full knowledge and consent of the town authorities for several years before I came to West Point." All this, of course, has now been altered.

### MEDICAL AIR SERVICE.

Special provision for the conveyance of medicines and surgical apparatus by aeroplane has been made by the Siamese Government, and much reduced rates for machines for the conveyance of doctors to areas where disease may break out are offered. Medicines and apparatus to be used for combating any outbreak are to be sent by air free of all charge beyond the usual postal tariff. In the event of an outbreak of disease among human beings or even animals the hire of a machine for rushing a doctor or a veterinary surgeon to the spot or from one district to another is about 2s. per mile. If a machine is needed for an errand of mercy the authorities may commandeer one.

### SMOKE ABATEMENT.

Under the direction of the Health Committee of Wakefield and Mr. W. H. Casmey, a Bradford engineer, says the *Manchester Guardian*, a demonstration was held at the works of Messrs. Paton & Baldwin, at Wakefield, when the chimney was seen "producing black smoke and no smoke at all at will. 'Let there be black smoke,' said Mr. Casmey, and there was, because that was virtually a command to the stoker to ignore all the lessons he had received from Mr. Casmey, and handle his fire carelessly. When, however, the stoker was enjoined to put an end to the smoke he was able, within a few minutes, to do so. There is any amount of support for Mr. Casmey's view that an instructed stoker can stop the emission of smoke, and at the same time economise fuel." The Wake field Health Committee have arranged classes for stokers under Mr. Casmey, which have been attended by from 70 to 90 students.

